# Measuring the operational impact of digitized hospital records: a mixed methods study

**DOI:** 10.1186/s12911-016-0380-6

**Published:** 2016-11-10

**Authors:** Philip J. Scott, Paul J. Curley, Paul B. Williams, Ian P. Linehan, Steven H. Shaha

**Affiliations:** 1 Centre for Healthcare Modelling and Informatics, University of Portsmouth, Buckingham Building, Lion Terrace, Portsmouth, PO1 3HE UK; 2 The Mid Yorkshire Hospitals NHS Trust, Pinderfields Hospital, Aberford Road, Wakefield, WF1 4DG UK; 3 EDM Project Manager, Royal Brompton and Harefield NHS Foundation Trust, Sydney Street, London, SW3 6NP UK; 4 Basildon and Thurrock University Hospitals NHS Foundation Trust, Nethermayne, Basildon, Essex SS16 5NL UK; 5 Center for Public Policy and Administration, University of Utah, Salt Lake City, UT 84112 USA

**Keywords:** Medical records, Electronic health records, Efficiency, Organizational, User-Computer Interface

## Abstract

**Background:**

Digitized (scanned) medical records have been seen as a means for hospitals to reduce costs and improve access to records. However, clinical usability of digitized records can potentially have negative effects on productivity.

**Methods:**

Data were collected during follow-up outpatient consultations in two NHS hospitals by non-clinical observers using a work sampling approach in which pre-defined categories of clinician time usage were specified. Quantitative data was analysed using two-way ANOVA models and the Mann-Whitney *U* test. A focus group was held with clinicians to qualitatively explore their experiences using digitized medical records. The quantitative and qualitative results were synthesized.

**Results:**

Four hundred six consultations were observed. Using paper records, there was a significant difference in consultation times between hospitals (*p* = 0.016) and a significant difference in consultation times between specialties within hospitals (*p* = 0.003). Using digitized records there was a significant difference in consultation times between specialties within a hospital (*p* = 0.001). Excluding outliers, there was no significant difference between consultation times using digitized records compared with consultations using paper records in the same hospital, either at site (*p* > =0.285) or specialty level (*p* > =0.122). With digitized records at site A, two out of three specialties showed a significant increase in time spent searching computer records (*p* < =0.010, Δ = 01:50–07:10) and one specialty had a corresponding reduction in time spent searching paper records (*p* = 0.015, Δ = −00:28). Site B showed a notable increase in direct patient care (*p* < 0.001, Δ = 04:20–06:00) and time spent searching computer records (*p* < =0.043, Δ = 00:10–01:40) and reductions in the other time categories.

The focus group confirmed that the most recent clinical letter was a vital document in the patient record, often containing most of the required information. Concerns were expressed about consistency of scanning practice, causing uncertainty about what could be relied upon to exist in the digitized record. Benefits of digitized records included: access from multiple locations, better prepared ward rounds, improved inpatient handovers and an improved timeline of patient events. Limitations of digitized records included: increased complexity of creating a patient summary, display of specialised content such as hand-drawn diagrams, inability to quickly flick through the pages to find relevant content.

**Conclusions:**

Digitized medical records can be implemented without detrimental operational impact. Inherent differences between specialties can outweigh the differences between paper and digitized records. Clear and consistent operational processes are vital for the reliability and usability of digitized medical records. Divergent views about usability (such as whether patient summary information is better or worse) may reflect familiarity with features of the digitized record.

## Background

Many hospitals have seen the use of digitized medical records (scanned paper) as a means to save money on administration and improve access to records [1, 2]. In the United Kingdom (UK), Government policy has repeatedly promoted the move away from paper records in health care [3]. However, published UK experience has shown that clinical usability of the digitized hospital record can be poor and potentially have negative effects on operational processes [4]. Even full electronic patient records (EPRs) have had detrimental impact on clinical productivity, both in the USA [5] and recent UK implementations [6, 7].

Hence, we believe that robust data is needed to determine if digitized hospital records can be implemented in a clinically acceptable way without detrimental operational impact within the UK National Health Service (NHS). Despite the volume of health informatics literature [8], there remains insufficient published research about digitized hospital records. To date, most of the published implementation experience about using digitized hospital records have been from projects in Norway [9–13].

For clarity, we first define our understanding of electronic health record acronyms. Electronic patient records (EPRs) [14] are also widely called electronic health records (EHRs) [15] or electronic medical records (EMRs) [16]. An influential NHS information strategy [17] attempted to distinguish the EPR from the EHR, with the former defined as a record maintained by a single healthcare institution and the latter as a longitudinal cradle-to-grave patient record drawn from multiple EPRs. However, in general usage the terms lack such precision and are often interchangeable. The HL7 EHR-System Functional Model defines an EHR as “a comprehensive, structured set of clinical, demographic, environmental, social, and financial data and information in electronic form, documenting the health care given to a single individual” [18]. Alternatively, ISO TR 20514:2005 defined a ‘basic generic’ EHR as simply a “repository of information regarding the health status of a subject of care, in computer processable form” [19].

The data within an EHR typically includes both structured and unstructured content. Structured data is usually directly typed or dictated into the system or received by electronic transfer (such as laboratory results) and is characterised by defined records, fields and coding schemes. In contrast, unstructured data includes items such as free text notes or scanned correspondence [20]. Any data can be coded using code systems and terminologies such as LOINC or SNOMED-CT, though obviously more structured data can be coded at a finer level of detail and hence offers more sophisticated analysis capabilities [21].

A digitized medical record is a paper record scanned into a set of unstructured computerized images, with some level of structured metadata for navigation and analysis purposes. This can exist in a standalone application or as a module of an EHR. There is a spectrum of capabilities and limitations in digitized medical record applications, from simple image display to complex tools for search, navigation or annotation. Software applications for digitized medical records are often seen as a form of electronic document management (EDM) and some publications and EHR vendors use this acronym to describe such functionality. Although document scanning is also widely used in UK primary care, the scope of this paper is digitized *hospital* records. We hypothesised that using digitized medical records would make a significant difference to outpatient consultation times and to the duration of tasks within a consultation.

## Methods

### Aims

The purpose of this study was to measure the effects of digitized medical records on the duration and time utilization of follow-up outpatient consultations as the measure of operational impact. The study compared timings between consultations using paper records with consultations using digitized records. We were not comparing one EDM system against another, but comparing any digitized record against standard paper-based practice. The study also sought the views of clinicians about the benefits and disadvantages of using digitized records. We selected follow-up rather than initial outpatient visits as the unit of measure, as some patients would have no pre-existing hospital medical record at their first consultation. The defined research questions and their purpose are listed in Table 1.

**Table 1:** Research questions

RQ	Question	Rationale
1	Does the duration of consultations using paper records vary between Trusts?	To understand the baseline variation in standard clinic operation by Trust and specialty.
2	Does the duration of consultations using paper records vary between specialties within a Trust?	
3	Does the duration of consultations using digitized records vary between Trusts?	To understand the variation in clinic operation using digitized records by Trust and specialty.
4	Does the duration of consultations using digitized records vary between specialties within a Trust?	
5	Does the duration of consultations using digitized records vary from consultations using paper records in the same Trust?	To determine the operational impact of implementing digitized records by Trust and specialty.
6	Does the duration of consultations using digitized records vary from consultations using paper records within specialties in the same Trust?	
7	Does task duration in consultations using digitized records vary from consultations using paper records within specialties in the same Trust?	

### Settings

The study was conducted in two English NHS Trusts: Mid Yorkshire Hospitals NHS Trust (MYHT) and Basildon & Thurrock University Hospital NHS Foundation Trust (BTUHT). Both of the Trusts were implementing digitized medical records (using different systems) and agreed to participate in the study to evaluate the operational impact. This study had no external funding, but data collection was resourced from within the Trusts’ implementation project budgets. Data were collected at each site within a few months of initial implementation (we use the terms “site” and “Trust” interchangeably), whilst clinics were still using a mixture of paper and digitized records.

MYHT specialties were gynaecology, paediatrics and vascular surgery, and BTUHT specialties were gynaecology, paediatrics and rheumatology. Clinical specialties for observation were selected for a combination of reasons. Primarily this was to include specialties that rely heavily on detailed patient information including history and prior findings and interventions, and are therefore impacted more substantially by availability of the digitized medical record. We were also constrained by practical logistics (based on where and when the digitized medical records were implemented) and our aim to allow some inter-site comparison of the same disciplines. This partly opportunistic approach produced an unbalanced design, but we took account of this in the analysis.

### Study design

The study emulated the approach of previous research into time effects of EHRs [22, 23]. Time sampling data were collected by a non-clinical observer using a work sampling approach in which pre-defined categories of clinician time usage were specified. The work sampling method is explained in the cited EHR time effect studies and cognate reports such as Munyisia, Yu & Hailey and Ammenwerth & Spotl [24, 25]. Data were gathered as they occurred naturalistically without randomization or blinding, thus representing a quasi-experimental approach [26]. The initial time categories were derived from previous work [22, 23], but some revisions were made by the research group to better suit the study context. The defined categories for clinician time usage are shown in Table 2.

**Table 2:** Outpatient consultation time categories

Code	Category	Example
A	Direct patient care	Talking to patient, physical examination.
B	Information searching (paper)	Looking for information in paper record before or during patient consultation. Checking details from paper record before or during patient consultation.
C	Information searching (computer)	Looking for information in computer systems before or during patient consultation. Checking details from computer systems before or during patient consultation.
D	Recording information (paper)	Making notes, completing forms.
E	Recording information (computer)	Typing, completing electronic forms.
F	Dictation	
G	Third party conversation	Teaching junior doctor, phone calls, advising colleagues.
H	Other (specify)	

For purposes of informed consent, an information sheet explaining the research was provided for each patient in each consultation, and the clinician explained that he or she (not the patient) was the subject of the study and that participation was optional. If either a patient or the clinician declined participation for any patient-clinician interaction then the observer would leave the room until the next patient was seen. In all instances of data gathering the observer was entirely passive and had no interaction with clinicians or patients during consultations and no patient data were collected during any observation. As this study was unfunded, a purely manual data collection method was employed rather than, for example, a digital camera to record consultations and timestamp activities. The observers employed a paper tally sheet and a stopwatch. The tally sheet contained instructions about how to categorize the time if a clinician was doing two things at once. So, for example, the instructions said “If also talking to patient, record as writing”. Time recorded as category A was only when the physician was doing nothing else. Due to the concurrent usage of digitized and paper records, and the use of multiple computer systems for other purposes such as diagnostic imaging and laboratory test requesting and reporting, there were both paper-based and computer-based consultations occurring in the same clinics.

At MYHT there was a single quantitative data collection in February 2011 (shown as Stage 1 in Table 3), comprising a mixture of paper-based consultations and those using digitized medical records, and a qualitative focus group held in June 2012 to explore their experiences using digitized medical records. At BTUHT there were two quantitative data collections, the first between December 2010 and January 2011 (shown as Stage 1 in Table 3) and the second in December 2012 (shown as Stage 2 in Table 3). The field notes were qualitatively analysed. The study design received a favourable opinion from an NHS research ethics committee in November 2010.

**Table 3:** Summary of observational data sets

Specialty	Stage 1		Stage 2		Totals	
	MYHT		BTUHT		Paper	Digitized
	Paper	Digitized	Paper	Digitized		
Gynaecology	27 (5)	11 (4)	68 (3)	50 (3)	95 (8)	61 (7)
Vascular surgery	49 (3)	11 (3)	0 (0)	0 (0)	49 (3)	11 (3)
Paediatrics	50 (5)	5 (1)	43 (3)	49 (3)	73 (8)	54 (4)
Rheumatology	0 (0)	0 (0)	43 (2)	0 (0)	63 (2)	0 (0)
Total	126 (13)	27 (8)	154 (8)	99 (6)	280 (21)	126 (14)

### Statistical analysis

The outcome variables were the duration of each follow-up outpatient consultation and the time spent on each activity category. In order to establish expected time parameters and perform power analyses for needed sample sizes, data from three published studies [27–29] were combined with anecdotal data (personal communications, August 2010) to estimate a coefficient of variation (standard deviation ÷ mean) for outpatient consultations. This was in the range 0.21–0.29 (mean 0.24). This gave a range of sample sizes from 33 to 92 per group to detect a 2-min difference (α = 0.05, β = 0.2). The mean coefficient of variation was used to estimate sample sizes on the standard follow-up consultation times recommended by the professions [30, 31]. A hypothetical 15-min consultation would need a sample size of 52 per group. The only relevant data source for the time spent searching/reading medical records was a simulation conducted in one of the Trusts in the study. Data from this produced a sample size of 25 per group. As the same observation would collect both outcome variables, the maximum sample size (52 consultations per specialty) was considered optimal. IBM SPSS® Version 22 was used to perform standard parametric and non-parametric tests.

Raw data were thoroughly reviewed prior to analysis to ensure that the most appropriate statistical techniques were applied. Consultation times were analyzed, both the entire time spent for each patient and the breakdown of time spent on each category: direct patient care, information searching on paper or computer, information recording and dictation. Two-way ANOVA models were used for research questions 1–6 and the Mann-Whitney *U* test was used for research question 7 due to the severe departure from normal distribution and homogeneous variance. As the units of analysis were different for research questions 1–4 and research questions 5–6, we used different ANOVA models. For research questions 1–4, we split the data file by record type and ran a two-way ANOVA with total consultation time as the dependent variable and site and specialty as the independent variables. For research questions 5–6, we split the data file by site and ran a two-way ANOVA with total consultation time as the dependent variable and record type and specialty as the independent variables. In both cases, we used a Type IV model due to the unbalanced design. For research questions 1–4, boxplots revealed three outlying data points. These were filtered out in the ANOVA model so that the variance was homogeneous, as required for this test. For research questions 5–6, more outliers were discovered. It was necessary to filter out cases where the total consultation time was greater than 30 min (*n* = 31) to achieve a data set with homogeneous variance (*n* = 375). For research question 7, we split the data file by site and specialty ran a Mann-Whitney *U* test with the time categories A-H as the dependent variables and record type as the independent variable. Given the relatively small sample sizes, we selected the exact computation method.

## Results

### Quantitative data summary and characteristics

Altogether 406 consultations were observed; shown by site, specialty and record type in Table 3. No observations were declined by the patient or clinician. The figure in brackets is the number of clinicians observed in each subgroup. The entire MYHT data set was gathered by one observer. The BTUHT data was collected by two observers in stage one and a third observer in stage two. The sample sizes achieved were lower than the target levels due to time and resource constraints within the Trust implementation projects.

### Total consultation duration

**Fig. 1:**
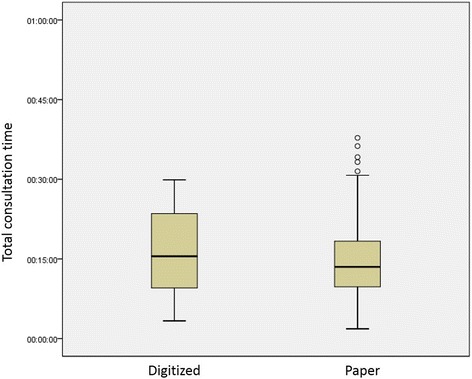
Distribution of total consultation time observations by record type (MYHT)

**Fig. 2:**
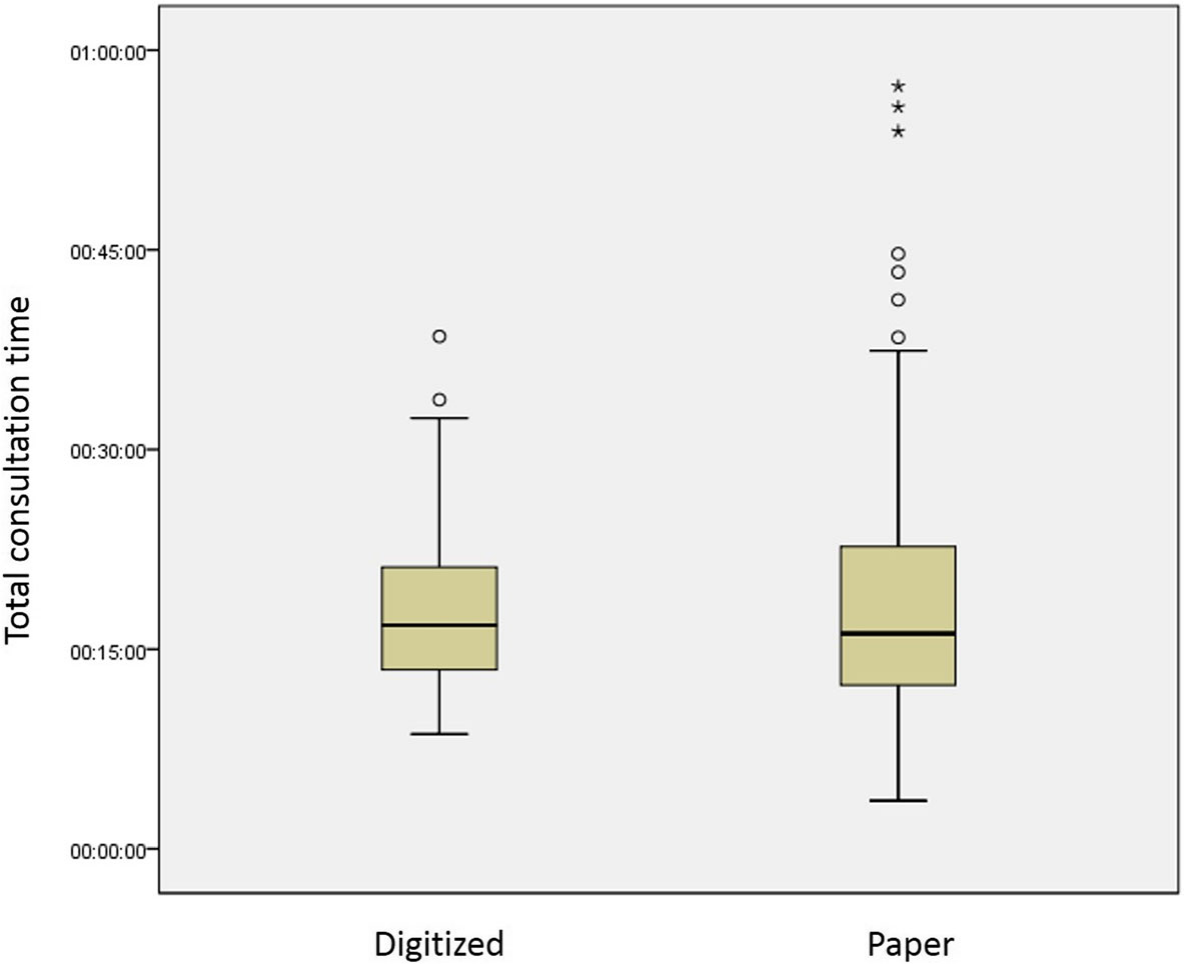
Distribution of total consultation time observations by record type (BTUHT)

Figures 1 and 2 illustrate the distribution of duration times as SPSS boxplots. Outliers are included for completeness, but some were excluded from analyses as explained above.

Table 4 shows the mean total consultation times, rounded to the nearest second, along with its 95 % confidence interval. The issue of potential measurement error is discussed later.

**Table 4:** Mean total consultation times by Trust and specialty^a,b^

Specialty	MYHT		BTUHT	
	Paper	Digitized	Paper	Digitized
	Mean (CI)	Mean (CI)	Mean (CI)	Mean (CI)
Gynaecology	15:05 (12:09–18:01)	15:27 (10:41–20:13)	17:19 (15:21–19:17)	15:53 (14:33–17:13)
Paediatrics	16:53 (14:50–18:56)	24:31 (19:49–29:13)	20:33 (17:39–23:27)	19:21 (17:39–21:03)
Rheumatology	-	-	16:16 (14:14–18:18)	-
Vascular surgery	12:29 (10:33–14:25)	12:25 (8:20–16:30)	-	-
Overall	14:47 (13:29–16:05)	15:54 (12:48–19:00)	17:53 (16:35–19:11)	17:36 (16:29–18:43)

Notes

^a^Blank cells where no data collected

^b^CI is the 95 % confidence interval of the mean

### Analysis

The ANOVA residuals were normally distributed, indicating that the results were reliable and readily interpretable. The results of analyses and interpretations are summarized in Table 5. Table 6 gives further detail of the significant differences between specialties found in the ANOVA models.

**Table 5:** Detailed ANOVA statistical results: Total consultation times^a^

RQ	Research question	Results	Interpretation
1	Does the duration of consultations using paper records vary between Trusts?	MYHT (mean = 14:47) significantly different from BTUHT (mean = 17:53), Δ = 03:06 (CI: 00:32–05:21), *p* = 0.016.	Viewed overall, there is a significant difference in consultation times between Trusts using paper records.
2	Does the duration of consultations using paper records vary between specialties within a Trust?	Specialties vary significantly within Trusts, *p* = 0.003. See Table 6 for details.	There is a significant difference in consultation times between specialties within a Trust using paper records.
3	Does the duration of consultations using digitized records vary between Trusts?	MYHT (mean = 15:54) not significantly different from BTUHT (mean = 17:36), *p* = 0.166.	Viewed overall, there is no significant difference in consultation times between Trusts using digitized records. However, the sample of digitized records at MYHT was small (*n* = 27).
4	Does the duration of consultations using digitized records vary between specialties within a Trust?	Specialties vary significantly within Trusts, *p* = 0.001. See Table 6 for details.	There is a significant difference in consultation times between specialties within a Trust using digitized records.
5	Does the duration of consultations using digitized records vary from consultations using paper records in the same Trust?	Overall, MYHT consultations using digitized records vary significantly from consultations using paper records, *p* = 0.006. However, excluding paediatrics (digitized records *n* = 5), there is no significant difference, *p* = 0.935. Overall, BTUHT consultations using digitized records do not vary significantly from consultations using paper records, *p* = 0.285.	Viewed overall, there is a significant difference between consultation times in clinics using digitized records compared with clinics using paper records in one of the two Trusts. However, this result appears to be biased by a small sample of observations in a single specialty.
6	Does the duration of consultations using digitized records vary from consultations using paper records within specialties in the same Trust?	MYHT consultations using digitized records vary significantly from consultations using paper records in the same specialty, *p* = 0.049. Excluding paediatrics (digitized records *n* = 5), there is no significant difference, *p* = 0.122. BTUHT consultations using digitized records do not vary significantly from consultations using paper records in the same specialty, *p* = 0.685.	Viewed overall, there is no significant difference between consultation times in clinics using digitized records compared with clinics using paper records in the same specialty, apart from in paediatrics at one site.

Note

^a^Δ denotes net difference

**Table 6:** Inter-specialty differences^a,b,c^

Specialty	Mean difference (CI) from Trust mean			
	MYHT paper	BTUHT paper	MYHT digitized	BTUHT digitized
Gynaecology	NSD	NSD	NSD	−01:43 (−00:38, −02:50)
Paediatrics	02:06 (00:16, 03:52)	02:40 (00:29, 04:31)	08:37 (02:08, 11:59)	01:45 (00:38, 02:50)
Rheumatology	-	NSD	-	-
Vascular surgery	−02:18 (−00:31, −04:08)	-	−03:29 (−01:01, −09:04)	-

Note

^a^Blank cells where no data collected

^b^NSD denotes no significant difference from Trust mean

^c^CI is the 95 % confidence interval of the difference

### Time categories

**Fig. 3:**
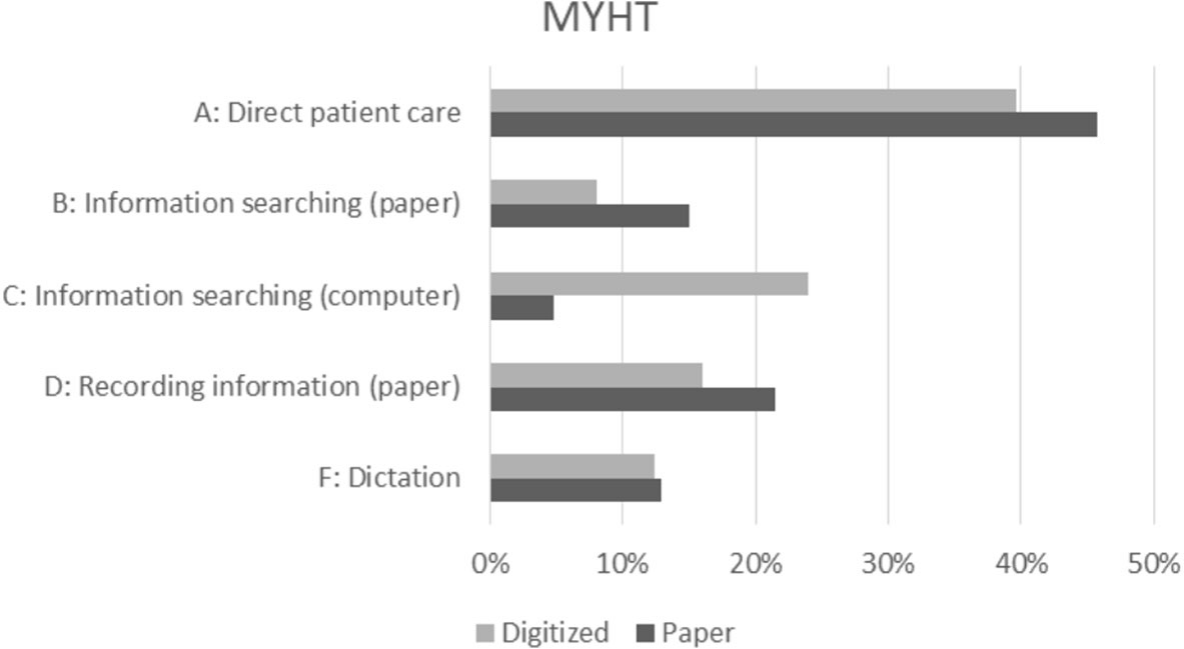
Median timings for task categories as % of consultation – MYHT

**Fig. 4:**
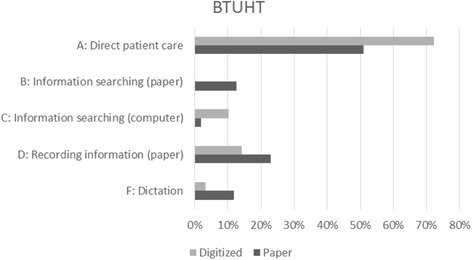
Median timings for task categories as % of consultation – BTUHT

Tables 7, 8 and Figs. 3, 4 summarize the median timings for each task category by site and specialty. The alphabetic codes refer to the time categories listed in Table 2 above.

**Table 7:** Median timings (mm:ss) for task categories – MYHT

	A	B	C	D	E	F	G	H
Paper								
Gynaecology	05:12	01:12	00:30	02:37	00:00	01:05	00:00	00:00
Paediatrics	05:25	02:30	00:00	03:35	00:00	01:25	00:00	00:00
Vascular surgery	04:14	00:55	00:55	00:52	00:00	01:20	00:00	00:00
Total	04:46	01:34	00:30	02:14	00:00	01:21	00:00	00:00
Digitized								
Gynaecology	04:33	00:45	01:00	01:50	00:00	01:00	00:00	00:00
Paediatrics	07:57	02:35	07:10	03:05	00:00	03:00	00:20	00:00
Vascular surgery	03:00	00:27	02:45	01:05	00:00	01:00	00:00	00:00
Total	04:33	00:55	02:45	01:50	00:00	01:25	00:00	00:00

**Table 8:** Median timings (mm:ss) for task categories – BTUHT

	A	B	C	D	E	F	G	H
Paper								
Gynaecology	05:08	01:26	00:00	03:49	00:00	01:20	01:08	00:00
Paediatrics	08:52	01:49	01:12	03:49	00:00	01:39	00:00	00:00
Rheumatology	07:14	01:47	01:19	01:23	00:00	01:33	00:00	00:00
Total	06:32	01:37	00:14	02:56	00:00	01:31	00:00	00:00
Digitized								
Gynaecology	09:28	00:00	01:40	02:03	00:00	00:57	00:00	00:00
Paediatrics	13:14	00:00	01:29	02:29	00:00	00:00	00:00	00:00
Total	11:23	00:00	01:37	02:14	00:00	00:30	00:00	00:00

Table 9 shows the results at specialty level contrasting the median times for paper and digitized records. Only significant differences are shown, with two-tailed p value and difference between median task category times.

**Table 9:** Task category median differences (mm:ss) for Tables 7 and 8 ^a,b^

	N	A	B	C	D	E	F	G	H
MYHT									
Gynaecology	38	NSD	NSD	NSD	NSD	NSD	NSD	NSD	NSD
Paediatrics	55	NSD	NSD	*p* = .004 Δ = 07:10	NSD	NSD	*p* = .03 Δ = 01:35	NSD	NSD
Vascular surgery	60	NSD	*p* = .015 Δ = -00:28	*p* = .010 Δ = 01:50	NSD	NSD	NSD	NSD	NSD
BTUHT									
Gynaecology	118	*p* < .001 Δ = 04:20	*p* < .001 Δ = -01:26	*p* < .001 Δ = 01:40	*p* = .001 Δ = -01:46	NSD	*p* = .014 Δ = -00:23	*p* < .001 Δ = -01:08	NSD
Paediatrics	92	*p* < .001 Δ = 06:00	*p* < .001 Δ = -01:49	*p* = .043 Δ = 00:10	*p* = .002 Δ = -01:20	NSD	*p* < .001 Δ = -01:39	NSD	*p* = .044 Δ: see text

Note

^a^NSD denotes no significant difference between paper and digitized

^b^Δ denotes net difference

At MYHT there was apparently a fairly predictable ‘swap’ between categories B (Information searching – paper) and C (Information searching – computer) in vascular surgery and a simple net increase in category C in paediatrics (though on a sample size of only 5, not too much can be made of this). Gynaecology showed no net effect on times per task category.

The situation at BTUHT was altogether more complex. Analyses were limited to the two specialties for which data were adequate. Both specialties showed significant differences in category A (direct patient care), B (information searching (paper)), C (information searching (computer)), D (recording information (paper)), F (dictation) and G (third party conversation). We suspect that the changes in F and G are random effects and are in any case clinically trivial as absolute time measures. Overall, notable increases in direct patient care and time spent searching the computer are the key findings, along with corresponding reductions in the other time categories. As shown in Tables 7 and 8, SPSS calculated the medians for category H (‘other’) as zero for each specialty although there were in fact 4/154 non-zero observations (range 00:28 to 01:24). The observer recorded these as interruptions of one kind or another. So the p value of the reported change for category H shown in Table 9 is statistically correct, though not operationally meaningful.

### Qualitative results

The focus group was held in June 2012 with nine clinicians from MYHT. The group comprised a cardiologist, two respiratory physicians, a paediatrician, a rheumatologist, two urologists, an anaesthetist and a vascular surgeon. Not all participants were present for the entire discussion.

Participants were asked about their experiences and perceptions regarding any possible impacts that scanned medical records have on clinical and operational activities. Several commented that the main hospital notes were often not yet fully scanned, so the first document they would look for would be the most recent clinical letter as a patient summary. When later asked how often that clinical letter contained most of the information needed, nearly half of answers ranged between 70 % and 90 %, while two said 50 % or less. One estimated that the clinical letter contained 25 % or less of the information needed, but all her patients were new referrals. Some participants observed that Emergency Department notes were now more accessible and reliably present in the record. Comments also noted that the overall standard of clinical letters had improved due to the increased reliance on them in the digitized record.

Some comments offered areas for improvement. The view was expressed that further guidance was needed to maximise the content value in notes, and that some departments were inconsistent in the structure and content of their letters. Others remarked that the operational scanning process varied between sites in the Trust, which led to uncertainty about what to expect within the digitized record. One participant commented about the legibility of handwritten text in digitized records, but also acknowledged that this was an issue with paper notes as well.

Another question asked the participants what they preferred about digitized records. Common answers related to the availability and accessibility of the record at multiple locations (including home) and the value this gave for off-site decision-making about patient care. Several participants noted how difficult care was before digitization when paper notes went missing: “We forget how it was when records did not turn up”. Two participants noted the utility of a feature of the digitized record application called the “timeline” which showed a summary of patient events in the record. One clinician observed that ward rounds were now quicker and that the nurses were better prepared, but that ward access still suffered from insufficient mobile hardware and some network issues. One clinician particularly noted the value of digitized records to support patient handovers: “Handovers morning and evening use scanned notes and PACS… I was a cynic but now I’m converted, especially for handovers. It is more intuitive than you think.”

The next question asked participants what they missed about paper-based notes. Comments included the ability to flick through notes easily, the comparative simplicity of creating a complete summary for medico-legal purposes or for patient transfers, and the display of medical photographs and hand-drawn diagrams. Divergent views offered about patient summary information may reflect varying familiarity with features of the digitized record. Alternatively, however, a real issue may be that abstracting the necessary data from each digitized document may be more difficult, even though having an additional summary timeline should represent an advantage.

When asked how their clinical time had been affected by digitized records versus paper, several said they perceived that clinics take longer. Others commented on the different approach needed for clinic preparation because there was no digital process analogous to paper-based preparation where a nurse would highlight relevant documents with sticky notes. Suggested solutions mentioned by respondents for making digitized processes better included keeping multiple windows open simultaneously for the same patient (such as PACS, digitized record, and laboratory test requests) and then previewing the various electronic sources for the next six patients so as to get a full overview. Overall, the general view seemed to be summed up by the comment that, “It is better practice but it takes longer”.

The final question asked whether respondents thought that, overall, the benefits of scanned records outweighed the disadvantages. Seven of nine participants said yes, and one said no.

## Discussion

Using paper records, there was a significant difference in mean consultation times between Trusts and a significant difference in consultation times between specialties within a Trust. This demonstrates a fundamental difference in standard practice both between sites and between specialties.

Aggregated at site level, there was no significant difference between mean consultation times using digitized records compared with consultations using paper records within the same Trust. This suggests that digitized records can be implemented without detrimental operational impact, when viewed at an overall Trust level. We found that differences between specialties are more pronounced than overall differences between sites or between paper and digitized records. Therefore the first part of our hypothesis, that using digitized records would make a significant difference to outpatient consultation times, was not supported.

### Differences in consultation duration between specialties

In our sample, differences between specialties outweighed differences between paper and digitized records. Earlier work by the first author has hypothesised that clinical specialty could be seen as a predictor of EHR acceptance [32]. Differences quantified in this study may reflect natural differences between specialties and specialists. This study appears to offer further support to the premise that, at the risk of gross over-simplification, time differences observed may in part reflect the varying “thinking styles” associated with practitioners and practices, wherein some disciplines are more narrative driven such as paediatrics, compared to other more ‘propositional’ specialties such as surgery.

When analysed by specialty, MYHT showed no significant difference in the duration of consultations using digitized records compared with consultations using paper records (see Table 4) except in paediatrics, where the mean consultation time was 17 min with paper records and 25 min with digitized records. However, it should be re-emphasized that only five consultations using digitized records were observed for paediatric clinics so the data may be unrepresentative. BTUHT showed no significant difference by specialty.

Despite these quantitative findings, the subjective perception expressed in the MYHT focus group was that ‘it takes longer’ with digitized records. This may be due to sampling differences – only two of the seven specialties represented in the focus group were also part of the quantitative study. It may also reflect a form of recall bias in respect of an initially unpopular change. Arguably, this further strengthens the case for *independent* evaluation of health IT interventions (rather than by implementers), so that a holistic evidence-based case may be made for whether and how they are adopted.

### Changes in time utilization

The small quantitative sample size for digitized records at MYHT is insufficient to draw conclusions at this level of detail. The larger data set from BTUHT offers some interesting findings about increased time in direct patient care. The second part of our hypothesis, that using digitized records would make a significant difference to the duration of tasks within a consultation, was supported for paediatrics and vascular surgery at MYHT and for paediatrics and gynaecology at BTUHT.

The BTUHT formal business case objectives included the need to deliver improvements in clinical efficiency and effectiveness – more generally recognised as measures for ‘releasing time to care’. The project took account of the usability lessons from other implementations where case notes have been scanned [4], recognising that the manner in which legacy scanned material is organised and indexed in the digitized record has a direct impact on the ease with which clinicians can find specific material. The three key components of the implementation that were designed to support clinical usability were:

1. The tab and sub-tab structure adopted for the digital record.
2. The identification of key-document-types within the physical record that can be individually identified within the scanned legacy material.
3. The association of document dates with a sub-set of the identified document-types.

Rich metadata can be associated with material that is generated ‘day-forward’ (post implementation and scanning of the legacy notes) and this helps create a structured, searchable digital record (the ‘future state’). However, associating metadata (document-type and document-date) with material in the *legacy* scanned record had significant cost implications for the project. Not all of the legacy scanned material could be indexed to the desired level of granularity. The implementation team therefore worked closely with a reference group of clinicians to select material in the physical case notes that was most appropriate for indexing. These discussions were augmented by a substantial number of direct observations by the project team of the way physical case notes are used in clinical settings. This helped to identify the material that was most commonly used in various outpatient clinical settings.

The digitized record was therefore effectively ‘tuned’ for clinical use – especially in outpatient clinics where it was known that there was significant pressure on clinical time. In addition, the use of ‘targeted indexing’ of the scanned legacy notes also meant that the ‘timeline’ functionality could be used to display the clinician-defined critical information for historic episodes. Digitising a clinical record removes the tactile and visual navigation pointers that help clinicians rapidly pinpoint information in the physical case note. However, by working closely with the clinical community, the project team was able to introduce digital markers – metadata – in the digitized legacy case note as simple and cost-effective navigation aids.

### User satisfaction

Laerum and colleagues reported lower clinician satisfaction with digitized images of records than with other components of an EHR [9, 11]. A follow-up study after three years [12] showed that user satisfaction with the digitized records in the EHR had remained roughly the same for medical secretaries, improved substantially for nurses and improved marginally for physicians. The ranking of user satisfaction remained unchanged: secretaries the highest, nurses somewhat below them and physicians the lowest of all.

These findings echo our qualitative data, where clinicians (physicians and surgeons in our sample) were not especially enthusiastic about scanned records but mostly agreed that, on balance, the disadvantages were less than the benefits – especially when viewed as a ‘package’ with other EHR benefits like electronic test ordering. Our clinical participants expressed particular concerns about the presentation of ‘special’ content such as hand-drawn diagrams and medical photographs. Our study did not probe the views of administrators or nurses so we cannot comment on that aspect.

### Workflow implications

Lium et al. noted that “old” routines built around paper records tended to persist even after the introduction of EPR and digitized records [13]. There are unavoidable workflow implications of moving either to digitized records or full EHR [33], but implementing what is *necessary* is not necessarily what is *optimal.* Furthermore, some workflow changes are planned and others are emergent.

Our qualitative data showed that the introduction of digitized records had unexpectedly led to improvements in the structure and content of clinic letters, as a contingency in the event of the full record being unavailable due to scanning delays. The majority of clinicians agreed that the latest clinic letter usually gave most of the information needed for the current patient encounter, except for new referrals.

Another workflow effect was the loss of ‘clinic preparation’, where a nurse would signpost particularly important elements in the paper record. In principle, there is no reason why a digitized record module could not support an analogous electronic process. However, this would need to take account of both digitized and natively electronic content so as to avoid the ‘paper-based thinking’ trap. It is easy to visualise some kind of electronic summary sheet for each patient where the nurse could drag files to create hyperlinks to particular documents, images and data to guide the physician into the consultation. We have not yet explored if such functionality is offered by commercial EHRs.

### Limitations

There might be a question as to whether each patient consultation within an overall series held as a clinic, with a notionally fixed endpoint, is a statistically independent event. We argue that clinical practice within the selected specialties was to treat each patient individually, and thus that each consultation is as long or as short as necessary. The wide variations seen in our data seem to support this inference. Other studies of general practice have treated patient consultations as statistically independent in a like manner [34, 35].

As this trial was not randomized and does not exactly compare like with like (specialty selection, timing, digitized record software), we cannot exclude confounding variables. For example, factors such as case mix, environmental features, secular trends and organizational or service changes, and the difference in the digital medical records between the two organisations, cannot be excluded. No obvious or large internal or external influences of these kinds were noted over the period of the data collections, so we are confident of the findings, but the limitation is acknowledged and the study cannot formally assert causality. We also accept that there is inevitably some unquantifiable effect from the timing of the quantitative data collection being within a few months of implementation rather than in a settled operational environment, and aspects of this were noted from our qualitative component. As we have not adjusted for multiple statistical testing, our conclusions should strictly be treated as exploratory not confirmatory [36].

Time categorization is another limitation worthy of mention. Time usage categorized as “Other (specify)” (category H) was zero, implying that the defined categories sufficiently captured the range of activity types. Category E, “Recording information (computer)” was zero too. This apparently because each hospital was continuing to take paper notes (on a form designed for immediate scanning) rather than using direct computerized data entry in clinics. Perhaps surprisingly, category G, “Third party conversation” (in effect, interruptions), was also zero in each data set except gynaecology at BTUHT. This seems to highlight a fundamental difference between the focussed and relatively undisturbed nature of clinical work in office-based clinics in contrast to the more challenging environment of inpatient wards or emergency departments [37, 38]. This difference is crucial for usability and adoption of digitized records and full EHRs [39].

The time-related data comprised relatively small samples. Each data set was uniquely collected by a single observer without any calibration or measure of intra- or inter-observer reliability, making measurement error the main weakness of this study, largely due to its unfunded nature. Additionally, although the data collection instrument was face validated, piloted and refined for ease of use, no measurement study was conducted on it. The quantitative data collection was resourced from existing NHS Trust project budgets using non-clinical observers recruited by the Trust project teams, therefore the risk of observer bias is acknowledged.

There has been considerable delay between data collection and publishing the results. As with several other factors, this is largely due to the unfunded nature of the project and hence the difficulty in resourcing the analysis and writing in competition with funded work. However, we believe there is an ethical duty to publish our findings given the paucity of work on this topic.

### Further work

We propose to undertake similar studies in other hospitals, with more robust measurement methodology and standardization, along with undertaking intra-observer and inter-observer reliability before and during the study [40]. We also aim to explore the use of the online tool TimeCaT [41].

An important question for digitized records, for which we currently have only anecdotal data, is finding the right balance between scanning cost and detailed document indexing (for ease of searching). Most importantly, we did not compare paper and digitized records with fully structured EHRs. EHR-based research may in fact make digitization definable as a step in transition from paper to the fully technologically advanced solution with inherent clinical decision support capabilities in addition to the accessibility benefits found with digitization. We also propose that a comparison of the underlying or concurrent EHR solutions within varying organisations is a future undertaking worthy of execution.

## Conclusions

The quantitative data we have reported suggests that digitized medical records can be implemented without substantial detrimental operational impact, and that inherent differences between specialties may outweigh the differences between paper and digitized records. The qualitative data stress the importance of clear and consistent operational processes to support and optimize the reliability and usability of digitized medical records. Further work is needed to compare digitized record performance with a structured and interactive EHR.
